# Identification of the Structural Features of Guanine Derivatives as MGMT Inhibitors Using 3D-QSAR Modeling Combined with Molecular Docking

**DOI:** 10.3390/molecules21070823

**Published:** 2016-06-23

**Authors:** Guohui Sun, Tengjiao Fan, Na Zhang, Ting Ren, Lijiao Zhao, Rugang Zhong

**Affiliations:** Beijing Key Laboratory of Environmental & Viral Oncology, College of Life Science and Bioengineering, Beijing University of Technology, Beijing 100124, China; sunguohui@emails.bjut.edu.cn (G.S.); fantengjiao2014@emails.bjut.edu.cn (T.F.); nanatonglei@bjut.edu.cn (N.Z.); renting@bjut.edu.cn (T.R.); lifesci@bjut.edu.cn (R.Z.)

**Keywords:** MGMT, inhibitors, 3D-QSAR, CoMFA, CoMSIA, docking

## Abstract

DNA repair enzyme O^6^-methylguanine-DNA methyltransferase (MGMT), which plays an important role in inducing drug resistance against alkylating agents that modify the O^6^ position of guanine in DNA, is an attractive target for anti-tumor chemotherapy. A series of MGMT inhibitors have been synthesized over the past decades to improve the chemotherapeutic effects of O^6^-alkylating agents. In the present study, we performed a three-dimensional quantitative structure activity relationship (3D-QSAR) study on 97 guanine derivatives as MGMT inhibitors using comparative molecular field analysis (CoMFA) and comparative molecular similarity indices analysis (CoMSIA) methods. Three different alignment methods (ligand-based, DFT optimization-based and docking-based alignment) were employed to develop reliable 3D-QSAR models. Statistical parameters derived from the models using the above three alignment methods showed that the ligand-based CoMFA (Q_cv_^2^ = 0.672 and R_ncv_^2^ = 0.997) and CoMSIA (Q_cv_^2^ = 0.703 and R_ncv_^2^ = 0.946) models were better than the other two alignment methods-based CoMFA and CoMSIA models. The two ligand-based models were further confirmed by an external test-set validation and a Y-randomization examination. The ligand-based CoMFA model (Q_ext_^2^ = 0.691, R_pred_^2^ = 0.738 and slope *k* = 0.91) was observed with acceptable external test-set validation values rather than the CoMSIA model (Q_ext_^2^ = 0.307, R_pred_^2^ = 0.4 and slope *k* = 0.719). Docking studies were carried out to predict the binding modes of the inhibitors with MGMT. The results indicated that the obtained binding interactions were consistent with the 3D contour maps. Overall, the combined results of the 3D-QSAR and the docking obtained in this study provide an insight into the understanding of the interactions between guanine derivatives and MGMT protein, which will assist in designing novel MGMT inhibitors with desired activity.

## 1. Introduction

A number of alkylating agents, such as methylating agents (e.g., temozolomide, dacarbazine and procarbazine) and chloroethylating agents (e.g., carmustine, nimustine, lomustine and laromustine), are frequently used in the clinical treatment of malignant tumors [1,2,3]. These agents attack the O^6^ position of guanine in DNA and result in forming a series of O^6^-alkylguanine lesions, which are believed to be crucial DNA adducts related to the anticancer activity of chemotherapies. For example, O^6^-methylguanine (O^6^-MG) lesion is produced by temozolomide. O^6^-chloroethylguanine is generated by chloroethylnitrosoureas and subsequently rearranges to N1,O^6^-ethanoguanine, which further undergoes the second alkylation on the complementary cytosine to form a DNA interstrand crosslink [2,3,4,5]. The cytotoxic effects of anti-tumor alkylating agents are primarily derived from these DNA lesions [5,6,7]. However, a unique DNA repair enzyme, O^6^-methylguanine-DNA methyltransferase (MGMT), also called O^6^-alkylguanine-DNA alkyltransferase (AGT), can repair the O^6^-lesion of guanine by transferring the O^6^-alkyl groups to the active center at the Cys145 residue and restore normal DNA. The repair was demonstrated to result in drug resistance in tumor cells [3,4]. After accepting the lesion groups, MGMT is rapidly degraded by a ubiquitination-dependent proteolysis [8,9,10]. One MGMT molecule can only repair one lesion, so it is considered as a “suicide enzyme”. Previous studies indicated that there was an inverse relationship between the levels of MGMT expression and the sensitivity to O^6^-guanine alkylating agents [1,11,12,13,14]. The increasing of MGMT level correlates well with the enhancement of tumor resistance to these alkylating agents [4,12]. 

Since high expression of MGMT can cause strong resistance to the guanine O^6^-alkylating agents, a series of MGMT inhibitors were synthesized and were used as adjuvants to improve the chemotherapeutic effects [13,14,15,16,17,18,19,20,21,22,23,24,25,26]. Although numerous inhibitors have been described, only two compounds, O^6^-benzylguanine (O^6^-BG) and O^6^-(4-bromothenyl)guanine (O^6^-4-BTG), entered clinical trials [3,4,27,28,29]. Unfortunately, phase II clinical trials of the two inhibitors in combination with guanine O^6^-alkylating agents exhibited only limited response even though the nontoxicity of the inhibitors to the host was confirmed in early trials [27,28,29,30]. The severe myelosuppression induced by the two MGMT inhibitors limits the dose of alkylating agents given in the combination treatments, which finally leads to the failure of the chemotherapy [4]. Therefore, it is necessary to develop novel MGMT inhibitors with high efficacy and selectivity to cancer cells.

Three-dimensional quantitative structure activity relationship (3D-QSAR) describes the linkage between the structural features and the bioactivities of compounds and also points to suggestions for designing novel inhibitors of enzymes [31,32,33,34]. Comparative molecular field analysis (CoMFA) has become one of the most widely used 3D-QSAR methods in rational drug design since it was first introduced by Cramer et al. in 1988 [35]. Comparative molecular similarity indices analysis (CoMSIA) is another widely used 3D-QSAR method, which describes the similarities and differences between ligands and correlates them with changes in the binding affinity by involving steric, electrostatic, hydrophobic and hydrogen-bond donor and receptor fields [36,37,38,39].

In this study, we built 3D-QSAR models for a series of guanine derivatives as MGMT inhibitors by CoMFA and CoMSIA analysis to reveal the relationship between the structural features of the substrates and the MGMT-inhibitory activity. A docking study was performed to gain insights into the binding interactions of the inhibitors and MGMT protein. This study will not only assist in the understanding of the mechanism of MGMT inhibition by guanine derivatives, but also provide useful information for designing novel MGMT inhibitors with desired activity.

## 2. Results and Discussion

### 2.1. Model Validation

The predictability and reliability of a 3D-QSAR model can be evaluated by checking several statistical parameters, including cross-validated correlation coefficient (Q_cv_^2^), non-cross-validated correlation coefficient (R_ncv_^2^), standard error of estimate (SEE) and F test value. For the chiral molecules in the training set, the *R*-isomers were used to construct the QSAR models. The QSAR models established using the *S*-isomers gave similar results (see Figure S1, Tables S1 and S2 in the Supplementary Materials). Table 1 lists the statistical parameters of the 3D-QSAR models constructed in this study. For the CoMFA analysis, the ligand-based model yielded a Q_cv_^2^ of 0.672, optimal number of principal components (ONC) of 8, R_ncv_^2^ of 0.997, SEE of 0.089 and F value of 1096.142. However, the other two CoMFA models derived from DFT optimization-based and docking-based alignments gave the Q_cv_^2^ and R_ncv_^2^ values below the standard of an eligible model.

**Figure 1:**
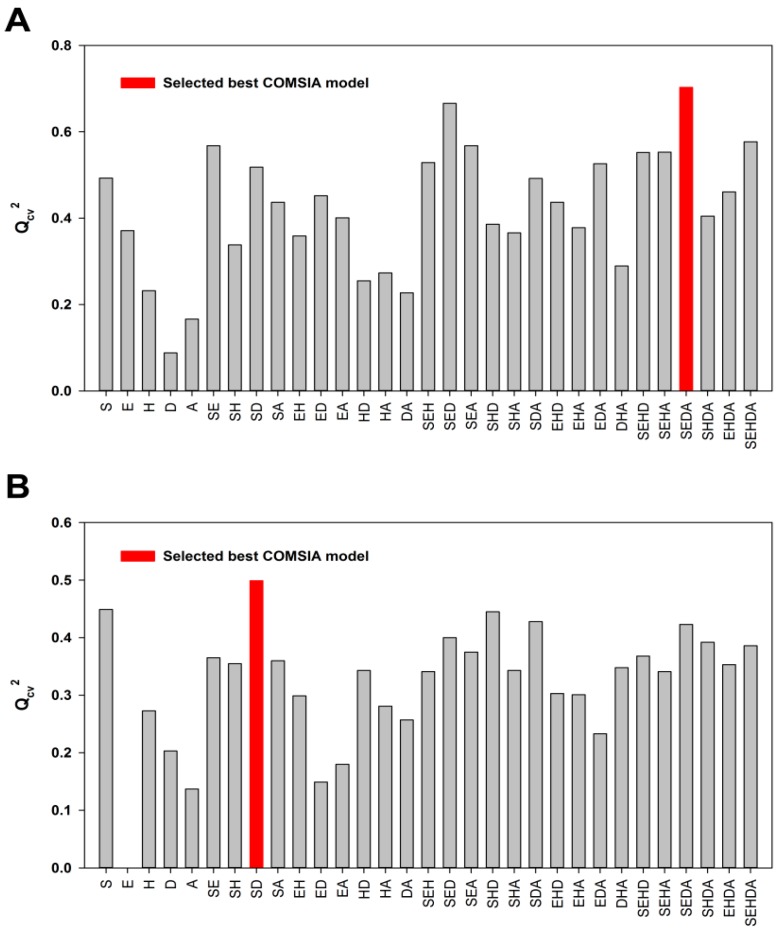

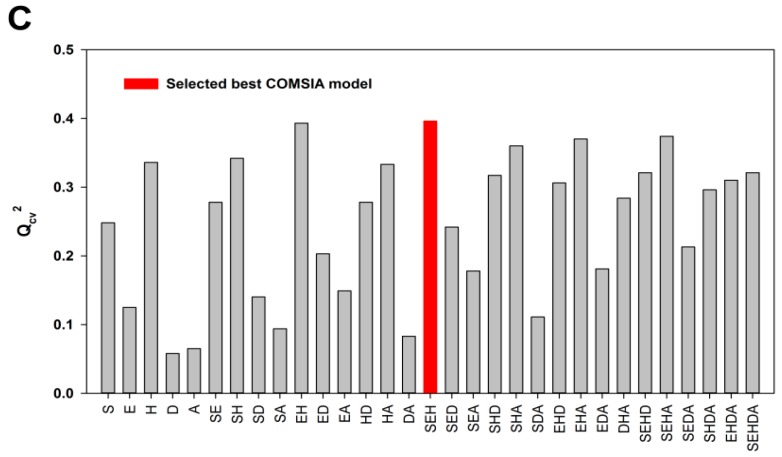
The statistical results of the possible field combinations. (**A**) The Q_cv_^2^ values of the ligand-based CoMSIA models; (**B**) The Q_cv_^2^ values of the DFT optimization-based CoMSIA models; (**C**) The Q_cv_^2^ values of the docking-based CoMSIA models.

Since the five CoMSIA descriptor fields—namely steric (S), electrostatic (E), hydrophobic (H), hydrogen bond donor (D) and acceptor (A)—are not totally independent from each other, different combinations of the five fields were used to obtain the best model [34,40,41]. The results of the 31 possible field combinations were shown in Figure 1 and the Q_cv_^2^ value was used to assess the statistical qualities of these 3D-QSAR models. The SEDA, SD and SEH field combinations with the highest Q_cv_^2^ values were selected for generating the best CoMSIA models using ligand-based, DFT optimization-based and docking-based alignment methods, respectively. Similar to the CoMFA model, the ligand-based CoMSIA model generated from the SEDA field combination gave a Q_cv_^2^ value of 0.703 and R_ncv_^2^ value of 0.946 that satisfies the statistical criterion of Q_cv_^2^ > 0.5 and R_ncv_^2^ > 0.9. The CoMSIA models derived neither from the DFT optimization-based alignment nor from the docking-based alignment meet the statistical criterion (Table 1). Therefore, only the CoMFA and CoMSIA models obtained from the ligand-based alignment were employed for further validation.

### 2.2. External Test Set Validation and Y-Randomization Test

**Figure 2:**
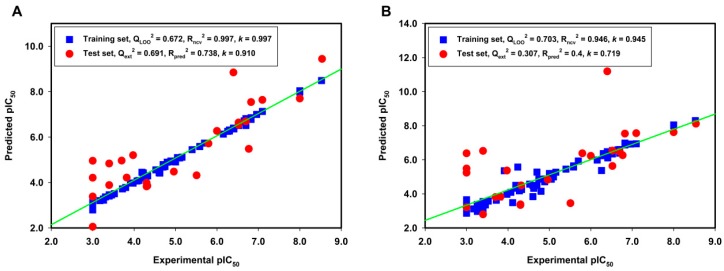
The linear correlation between the experimental and predicted pIC_50_ values for the training set (blue square) and the test set (red circle) based on (**A**) the CoMFA model and (**B**) the CoMSIA model derived from the ligand-based alignment method.

Although the CoMFA and CoMSIA models derived from ligand-based alignment were both observed with Q_cv_^2^ > 0.5 and R_ncv_^2^ > 0.9, a 3D-QSAR model with acceptable predictability also requires to meet other statistical criterions, including external validation correlation coefficient (Q_ext_^2^ **>** 0.5), predictive correlation coefficient (R_pred_^2^ > 0.6) and slope *k* (0.85 ≤ *k* ≤ 1.15) [42]. So, a test set containing 25 compounds independent from the training set was used for an external validation to confirm the predictability of the obtained CoMFA and CoMSIA models. Table 2 lists the predicted pIC_50_ values of the training and the test sets, as well as the residues between the experimental and predicted pIC_50_ values. The linear correlations between the experimental and predicted pIC_50_ values for the CoMFA and CoMSIA models were shown in Figure 2A,B, respectively. The Q_ext_^2^, R_pred_^2^ and *k* values are 0.691, 0.738 and 0.91 for the CoMFA model, respectively; and are 0.307, 0.4 and 0.719 for the CoMSIA model, respectively. A few outliers, such as compounds **82** and **91** in the test set, were observed with comparatively high residues between the experimental and the predicted activities. There are two possible reasons that may account for the failure of the models in outliers. Firstly, limited structural information on the C8 position of guanine (compound **82**) can be obtained from the 3D-QSAR models. Secondly, there is a unique structural difference of R_1_ group in compound **91** when compared to the other guanine derivatives in the training set. The results of the external validation using the test set suggested that the CoMFA model was more satisfying than the CoMSIA model derived from ligand-based alignment method.

A Y-randomization test was also performed to evaluate the possibility of the chance correlation in the CoMFA model [43]. The dependent variables (pIC_50_ values) were randomly shuffled and new QSAR models were constructed using the original independent variable matrix. If the QSAR models obtained by shuffling the pIC_50_ values gave lower Q_cv_^2^ values than the original model, we considered that the CoMFA model was not affected by any chance correlation. As shown in Table 3, none of the Q_cv_^2^ values was higher than 0.3 for 15 tests, which further indicated that the resulting CoMFA model derived from the ligand-based alignment was robust.

### 2.3. 3D Contour Map Analysis

**Figure 3:**
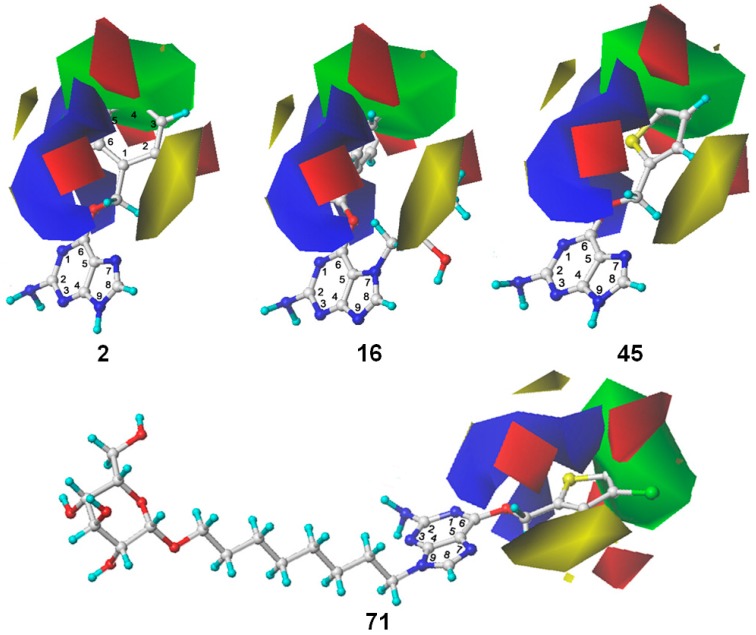
The CoMFA STDEV*COEFF contour maps for compounds **2**, **16**, **45** and **71**. The green and yellow region represent the sterically favorable and unfavorable properties, respectively. The blue and red region represent the electropositive and electronegative favorable properties, respectively.

The information visualization by 3D contour maps is an attractive feature of 3D-QSAR modeling, which can provide information about how to increase or decrease the biological activity of the investigated compounds. Different colors in 3D contour maps help to understand the relationship between the diversified steric and electrostatic field related to the activity of the compounds. As shown in Table 1, the steric and electrostatic fields account for 55.3% and 44.7% of the field contribution, respectively. Figure 3 displays the steric and electrostatic contour maps of the resulting CoMFA model. The steric field was presented in green and yellow colors, and the electrostatic field was shown in blue and red colors.

For the steric field, the green and yellow regions represent the sterically favorable and unfavorable properties, respectively. A yellow region was observed around the N7 position of guanine, which suggested that the bulky substitution in this region was unfavorable for the inhibitory activity to MGMT. This explains the relatively low inhibitory activities of compounds **14**–**16** and **30** with -CH_2_COOCH_2_CH_3_, -CH_2_CONH_2_, -CH_2_CH(OH)CH_2_CH_3_ and methyl groups, respectively, at the N7 position when compared to compound **2** without any N7-substituent group. Another yellow region near the ortho-position of the benzene ring of compound **2** suggested that bulky substitution in this region also contributed to the decrease of the inhibitory activity. On the other hand, a big green polyhedron-like region was found around the C3´-C5´ positions of the benzene ring. This could be the reason why compounds **2**–**4**, **19**–**23**, **40** and **41** exhibited higher activities than compounds **42**, **43** and **44** with substituent groups on the ortho-position of benzene ring. Furthermore, the higher activities of compounds **2**–**4**, **19**–**23**, **40** and **41** than compounds **1**, **8** and **32** are also in accordance with this conclusion.

For the electrostatic field, the blue and red regions represent the electropositivity and electronegativity favorable properties, respectively. The major blue region was found at the left wing as shown in the reference molecules, which indicated that electropositive substituent groups in this region were favorable for high inhibitory activity. For example, compound **2** was observed with higher activity than compound **31** bearing an electronegative nitrogen atom in the pyridine group. Four red regions were located near the plane of the benzene ring of compound **2** and the thenyl ring of compound **45**. This explains the reason why most benzyl- or thenyl-substituted guanine derivatives were more potent than alkyl-substituted guanine derivatives. Moreover, it can be seen from the contour maps that the C8 and N9 position of guanine is relatively well tolerated. 

### 2.4. Docking Analysis

Molecular docking studies were performed to predict the binding mode of the inhibitors with MGMT protein using the GOLD Suite 5.2 software (Cambridge Structural Database System). The crystal structure of MGMT protein with PDB entry of 1QNT (1.9 Å resolution) was selected for the docking studies [44]. We conducted the docking for all 97 compounds including the training and the test sets. The pose of each compound was selected according to the fitness score and the orientation. The binding affinities of the compounds with the receptor were presented by the docking scores [45]. The detailed docking results are listed in Table 4. Most of the compounds, which were docked into the active pocket of MGMT protein, presented a similar conformation as the ligand in the crystal structure of MGMT (PDB entry: 1T38) and agreed with the repairing mechanism of MGMT [46].

**Figure 4:**
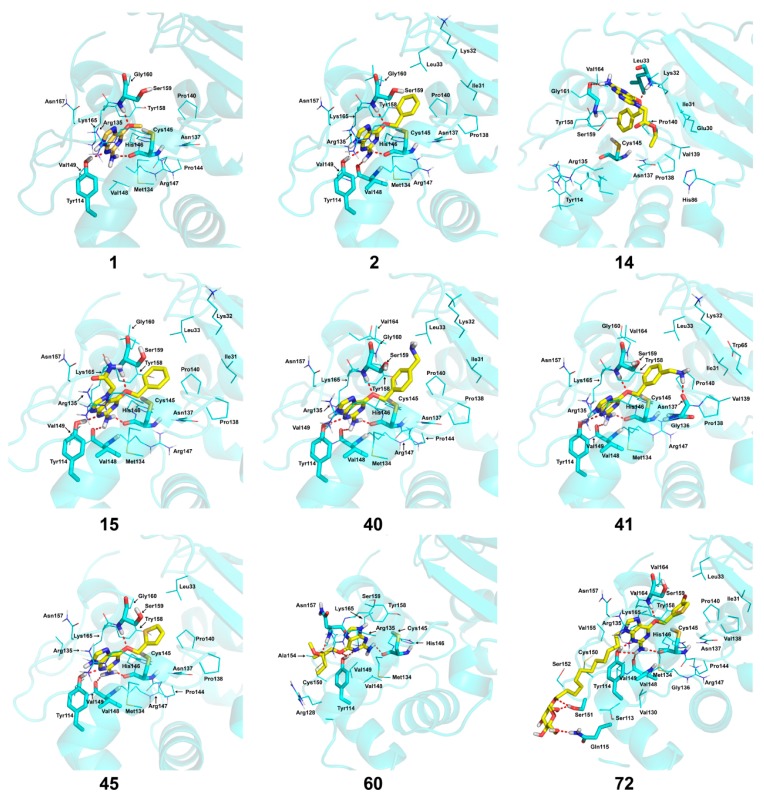
The optimal docking conformations of the representative molecules with MGMT protein (PDB entry: 1QNT). The protein is displayed as a cartoon model in cyan color. The ligands are depicted as stick models with yellow representing carbon atoms. The hydrogen bonds between ligands and receptor are represented by red dotted lines and the residues forming hydrogen bonds are presented in stick model with cyan for carbon atoms. The remaining residues in the active pocket are displayed as line models with cyan representing carbon atom. Nonpolar hydrogens were hidden. All figures were generated using PyMOL software (Educational version; www.pymol.org; DeLano Scientific, San Carlos, CA, USA).

Figure 4 shows the optimal docked conformations of several representative molecules (**1**, **2**, **14**, **15**, **40**, **41**, **45**, **60** and **72**) with MGMT protein. Three hydrogen bonds were formed between compound **1** (O^6^-MG) and the receptor, while four hydrogen bonds were formed between compound **2** (O^6^-BG) and the receptor. The resulting pose of **14** was far away from the active pocket of the receptor due to the steric effect. Although **15** can be docked into the active pocket, there is a strong steric clash between the N7-substituent group and residues Arg135 and Ser159. All N7-substituted guanine derivatives exhibited low binding affinities with the receptor, which accounted for the low inhibitory activities of compounds **14**–**16**, **30** and **75**. This is consistent with the 3D contour map analysis that a sterically unfavorable region was observed around the N7 position of guanine. By comparing compounds **2**, **40**, **41** and **45**, we found that there were four residues (Tyr114, Cys145, Val148 and Ser159) in the active pocket involved in the hydrogen-bonding formation of **2**, **40** and **45** with the receptor, whereas an additional hydrogen bond was formed between the –NH_2_ group of **41** and the oxygen atom of Asn137. Combined with the binding affinities, the results explain why the inhibitory activity follows the order of **45** > **41** > **40** > **2**. The higher potency of **40** and **41** than **2** was also supported by the 3D contour map where a big green polyhedron-like region was found around the C3´-C5´ positions of the benzene ring. It is worth noting that compound **60** displays an opposite orientation compared to the pose of the ligand in the crystal structure of MGMT (PDB entry: 1T38) [46], which leads to the loss of inhibitory activity. Compound **72** monosaccharide-conjugated on the N9 position forms eight hydrogen bonds with the Tyr114, Gln115, Cys145, Val148, Ser151 and Ser159 residues of MGMT protein, which results in the highly potent activity of **72** (pIC_50_ = 8.00). Figure S3 in the Supplementary Materials displays the ligand-binding surface of MGMT protein with the compounds described above, which helps to further visualize the docking results.

Besides, the formation of hydrogen bonds suggests that the -NH_2_ group on the C2 position of guanine is essential for high inhibitory activity, which explains the low activities of compounds **13**, **29**, **37**–**39**, **83**, **84**, **96** and **97** when compared to compound **2**. Similarly, the hydrogen bond formed between the O^6^ atom of guanine and Ser159 accounts for the higher inhibitory activity of compound **2** than compounds **17** and **18** with the O^6^ atom replaced by sulphur. Furthermore, a narrow space was found between the C2 or C8 atom of guanine and the active pocket of the receptor, suggesting large substituent groups not allowed in these sites. On the contrary, a wide entrance near the N9 position of guanine indicates that a bulky substituent group in this site is tolerated. Docking studies identified the key residues in the active pocket of the receptor such as Tyr114, Gln115, Arg135, Asn137, Cys145, Val148, Ser151, Tyr158 and Ser159. These residues are main contributors to the interactions between the inhibitors and MGMT protein.

## 3. Experimental Section

### 3.1. Data Set

**Figure 5:**
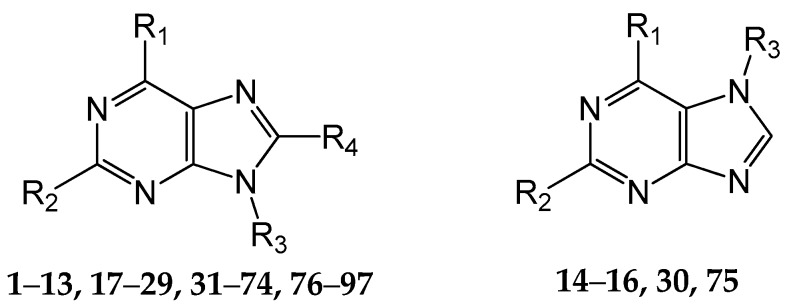
The general structures of all compounds used in this study.

A set of 97 guanine derivatives with different inhibitory activity against MGMT, which were chosen from literatures [17,18,19,22,23,25,26], were used as a data set for molecular modeling. The activities of all compounds were tested in vitro under the same experimental conditions in terms of half maximal inhibitory concentration (IC_50_) values. All original IC_50_ values were converted into the corresponding pIC_50_ values (pIC_50_ = −logIC_50_) and were used as the dependent variables in 3D-QSAR analysis. The pIC_50_ values for the data set range from 3.00 to 8.54, suggesting an adequate data collection for the 3D-QSAR study. The chemical structures and the pIC_50_ values for all compounds are listed in Table 5. Figure 5 dispalys the general structures for these compounds. The 97 compounds were randomly divided into two subsets, a training set including 72 compounds used for constructing the 3D-QSAR models and a test set including 25 compounds used for evaluating the external predictive ability of the models. Since the chirality of molecules **12**, **16**, **26**–**28**, **80** and **87** are unknown, we performed two parallel QSAR studies including the *R*-isomers and the *S*-isomers.

### 3.2. Molecular Modeling and Alignment

The 3D structures of all molecules in the data set were constructed using SYBYL 8.0 molecular modeling package (Tripos Inc., St. Louis, MO, USA). Energy minimizations were performed by Tripos force field [47] with Powell conjugate gradient descent method [48] and the partial atomic charges were added using Gasteiger-Huckel method [49]. The energy minimization was terminated when the energy gradient convergence criterion of 0.001 kcal/mol Å was reached and the maximum number of optimization steps was set to 1000.

**Figure 6:**
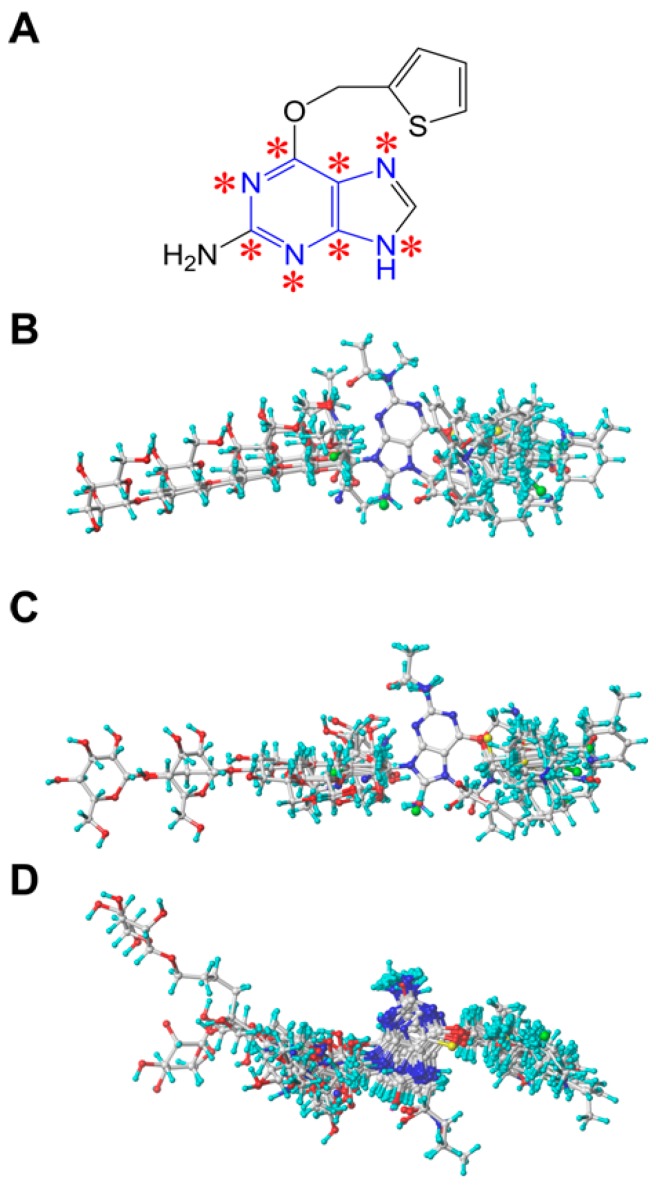
(**A**) Structure of the template molecule (compound **45**) used for the alignment. The common substructure atoms are marked with asterisks. Alignments of all molecules using (**B**) ligand-based, (**C**) DFT optimization-based and (**D**) docking-based alignment method.

The quality of 3D-QSAR models is usually sensitive to a specific alignment method [41,50]. In this study, three different alignment methods were employed to construct the 3D-QSAR models. Firstly, a ligand-based alignment (superimposition I) was used for the 3D-QSAR analysis. We chose compound **45** with the highest activity as a template to fit the remaining compounds of the training and test set using the “align database” function. The common substructure of the template molecule and the other molecules is depicted in Figure 6A with blue color. The resulting alignment conformations are shown in Figure 6B. Secondly, a DFT optimization-based alignment (superimposition II) was employed by performing geometry optimization on all molecules using the density functional theory (DFT) method with the B3LYP/6-31G+(d,p) basis set (GAUSSIAN-09 program package, Gaussian Inc., Wallingford, CT, USA) [51]. The 3D structures of all molecules were added with Gasteiger-Huckel partial atomic charges using SYBYL package and the alignment procedure is the same to superimposition I. The obtained alignment conformations are shown in Figure 6C. Thirdly, a docking-based alignment (superimposition III) was performed and the obtained alignment conformations were shown in Figure 6D. The active conformation of each compound was obtained from molecular docking by considering binding orientation and scoring. The selected conformation was added with Gasteiger-Huckel partial atomic charges followed by the alignment as described in superimposition I and II. The conformations of the inhibitors obtained from the three alignment methods are similar and agree with the repairing mechanism mediated by MGMT [5,44,46].

### 3.3. 3D-QSAR Studies

The 3D-QSAR models were constructed using CoMFA and CoMSIA methods. Steric and electrostatic potential fields for CoMFA were calculated at each lattice intersection of a regularly spaced grid of 2.0 Å. An sp^3^ hybridized carbon atom with a charge of +1.0 was used as the probe atom to calculate the CoMFA steric and electrostatic fields. The cut-off value was set to 30 kcal/mol. For CoMSIA analysis, in addition to steric and electrostatic fields, hydrophobic, hydrogen bond donor and acceptor fields were also considered. The descriptors of CoMSIA were calculated using the same lattice box as that employed in CoMFA calculations. The similarity indices of all the five fields (steric, electrostatic, hydrophobic, hydrogen bond donor and acceptor) were calculated using a sp^3^ hybridized carbon atom with a radius of 1.0 Å and +1.0 charge, +1.0 hydrophobicity, +1.0 hydrogen bond donor and +1.0 hydrogen bond acceptor properties. The attenuation factor was set to the default value of 0.3. A Gaussian function was employed to calculate the distance between the probe atom and each atom of the molecule.

A partial least squares (PLS) regression [52] was used to obtain statistically significant 3D-QSAR models. For PLS analysis, the CoMFA and CoMSIA descriptors were used as the independent variables, and the pIC_50_ values were used as the dependent variables. Leave-one-out (LOO) method was used to perform a cross-validation analysis, in which one molecule is removed from the data set and its activity is predicted by the model derived from the remaining molecules of the data set. Then, the cross-validated correlation coefficient (Q_cv_^2^) and the optimal number of principal components (ONC) were determined. After getting the ONC, a non-cross-validation analysis was performed to obtain the conventional correlation coefficient (R_ncv_^2^), standard error of estimate (SEE) and F value. Finally, the 3D-QSAR models were generated.

The test set of the compounds, which are not included in model generation, were used to evaluate the robustness and statistical significance of the 3D-QSAR models [42,43,53]. The pIC_50_ values of the test set were predicted based on the constructed models and then the predictive correlation coefficient (R_pred_^2^) was calculated using Formula (1) [38,42].

R_pred_^2^ = (SD-PRESS)/SD (1)

where SD is the sum of squared deviations between the activities of the test set molecules and the mean activity of the training set molecules. Predicted residual sum of squares (PRESS) is the sum of squared deviation between the predicted and the actual activity of each molecule in test set.

### 3.4. Molecular Docking

Molecular docking study was carried out using GOLD Suite 5.2 software. We selected the crystal structure of MGMT with PDB entry of 1QNT (1.90 Å resolution) [44] as a receptor for docking study. In order to validate the docking approach, self-docking was conducted using the X-ray crystal structure of human MGMT with PDB entry of 1T38 (3.2 Å resolution), which is a protein-ligand complex with MGMT bounding to DNA containing O^6^-MG. In the crystal structure of 1T38, the Cys145 residue was experimentally mutated to serine to avoid the covalent transferring of the methyl group on O^6^-MG [46]. For the self-docking, the protein-DNA complex model of 1T38 was simplified by removing the DNA double strands except for the O^6^-MG substrate and deleting the solvent molecules in the X-ray crystal. Hydrogen atoms were added to the protein and Gold score was chosen as a scoring function. The docking was performed by the “cytochrome P450 mode” in GOLD software and the active site was located at the Cys145 residue. The root mean square deviation (RMSD) of the docked pose was 0.0882 Å when compared to the pose in the crystal complex (see Figure S2 in the Supplementary Materials), which suggested that the docking conformation produced by GOLD closely resembled the crystal structure. Thus, GOLD is suitable for performing the docking of guanine derivatives to MGMT protein.

## 4. Conclusions

A 3D-QSAR study was performed based on a series of guanine derivatives as MGMT inhibitors using CoMFA and CoMSIA methods. Three different alignment methods were used to overlap the molecules. The optimal 3D-QSAR model was derived from CoMFA with the ligand-based alignment. The 3D contour maps provide crucial information of the steric and electrostatic field for the design of novel guanine derivatives with high MGMT-inhibitory activity. Molecular docking study was performed to explore the binding mode between the guanine derivatives and MGMT protein. The docking results suggest that the key residues in the active pocket of the receptor, including Tyr114, Gln115, Arg135, Asn137, Cys145, Val148, Ser151, Tyr158 and Ser159, play important roles in the interactions of the ligands and receptor. The oxygen atom at the C6 position and the -NH_2_ group at the C2 position of guanine are essential for high MGMT-inhibitory activity. The substituent groups on the N7 position of guanine are unfavorable for the inhibitory activity due to the steric effect. A substituent group with limited size is allowed for the C8 position of guanine, while the N9 potion of guanine is highly tolerated. The combined analysis of the 3D contour maps and the docking results provide valuable information for the further understanding of the structure–activity relationship of guanine derivatives as MGMT inhibitors, which will assist in designing novel MGMT inhibitors with high activity.

## Figures and Tables

**Table 1:** Statistical parameters of the CoMFA and CoMSIA models derived from three alignment methods.

Parameters ^1^	Ligand-Based Alignment		DFT Optimization-Based Alignment		Docking-Based Alignment	
	CoMFA	CoMSIA ^2^	CoMFA	CoMSIA ^2^	CoMFA	CoMSIA ^2^
Q_cv_^2^	0.672	0.703	0.498	0.499	0.164	0.396
ONC	8	13	4	4	4	5
R_ncv_^2^	0.997	0.946	0.717	0.695	0.696	0.763
SEE	0.089	0.384	0.814	0.846	0.845	0.751
F value	1096.142	77.545	42.483	38.108	38.273	42.425
Field Distribution (%)						
Steric	55.3	25.8	66.5	54.7	48.0	14.9
Electrostatic	44.7	45.0	33.5	–	52.0	46.0
Hydrophobic	–	–	–	–	–	39.1
HBD	–	13.4	–	45.3	–	–
HBA	–	15.8	–	–	–	–

^1^ Q_cv_^2^, ONC, R_ncv_^2^, SEE, F value, HBD and HBA are cross-validated correlation coefficient, optimal number of principal components, non-cross-validated correlation coefficient, standard error of estimate, F test value, hydrogen bond donor and hydrogen bond acceptor, respectively; ^2^ The parameters of CoMSIA models were derived from the combination of different fields that generates the highest Q_cv_^2^ value.

**Table 2:** Comparison of the experimental pIC_50_ values, predicted pIC_50_ values and residual values of the 97 compounds for CoMFA and CoMSIA models derived from the ligand-based alignment method.

Compounds	Experimental pIC_50_	CoMFA		CoMSIA	
		Predicted pIC_50_	Residues	Predicted pIC_50_	Residues
**Training Set**					
**1**	3.46	3.45	0.01	3.35	0.11
**2**	6.70	6.51	0.19	6.39	0.31
**3**	6.70	6.61	0.09	6.43	0.27
**4**	6.70	6.81	−0.12	6.60	0.10
**5**	5.70	5.73	−0.03	5.93	−0.23
**6**	5.00	4.91	0.09	5.19	−0.19
**7**	5.05	5.09	−0.05	4.86	0.19
**8**	4.70	4.68	0.02	4.49	0.21
**9**	4.52	4.56	−0.03	4.57	−0.05
**10**	4.89	4.91	−0.03	4.87	0.01
**11**	4.33	4.29	0.04	4.26	0.07
**12**	4.89	4.95	−0.06	4.71	0.18
**13**	4.07	4.06	0.01	4.09	−0.02
**14**	3.40	3.41	−0.01	3.38	0.02
**15**	3.40	3.42	−0.02	3.26	0.14
**16**	3.40	3.42	−0.02	3.55	−0.15
**17**	3.40	3.46	−0.06	3.07	0.33
**18**	3.40	3.40	−0.01	3.26	0.14
**19**	6.52	6.59	−0.07	6.34	0.18
**20**	6.30	6.27	0.03	6.36	−0.06
**21**	6.30	6.31	−0.01	6.24	0.06
**22**	5.40	5.45	−0.05	5.45	−0.05
**23**	6.52	6.51	0.01	6.35	0.17
**24**	6.40	6.40	0.00	6.47	−0.07
**25**	5.59	5.59	0.00	5.58	0.01
**26**	4.74	4.70	0.04	4.86	−0.12
**27**	3.97	3.97	0.00	3.98	0.00
**28**	5.15	5.11	0.04	5.27	−0.11
**29**	4.62	4.41	0.21	4.33	0.29
**30**	4.28	4.24	0.04	4.28	0.01
**31**	4.24	4.19	0.05	5.57	−1.33
**32**	3.52	3.51	0.02	3.58	−0.05
**33**	6.15	6.13	0.02	5.98	0.17
**34**	6.52	6.52	0.00	6.55	−0.02
**35**	7.10	7.13	−0.03	6.94	0.16
**36**	4.22	4.42	−0.20	4.33	−0.11
**37**	4.60	4.43	0.17	4.49	0.11
**38**	4.19	4.45	−0.26	4.50	−0.31
**39**	3.80	3.78	0.02	3.81	−0.02
**40**	6.82	6.85	−0.03	6.87	−0.05
**41**	6.96	7.00	−0.04	6.92	0.04
**42**	4.28	4.21	0.07	4.17	0.10
**43**	3.91	3.98	−0.07	5.35	−1.44
**44**	3.18	3.20	−0.03	3.12	0.06
**45**	8.52	8.49	0.03	8.30	0.22
**46**	3.00	3.01	−0.01	3.25	−0.25
**47**	3.00	2.90	0.10	2.99	0.01
**48**	3.31	3.365	−0.06	3.31	0.00
**49**	3.00	3.01	−0.01	3.12	−0.12
**50**	3.26	3.27	−0.01	3.37	−0.11
**51**	3.00	2.79	0.21	3.11	−0.11
**52**	4.60	4.50	0.10	3.84	0.77
**53**	3.26	3.22	0.04	2.97	0.29
**54**	4.80	4.89	−0.10	4.15	0.64
**55**	3.00	3.05	−0.04	3.65	−0.65
**56**	4.11	4.09	0.03	3.48	0.63
**57**	4.70	4.79	−0.09	5.28	−0.58
**58**	3.00	3.14	−0.14	2.96	0.04
**59**	3.00	2.96	0.04	2.86	0.14
**60**	3.00	3.00	0.00	3.34	−0.34
**61**	6.26	6.26	0.00	5.37	0.89
**62**	3.00	3.03	−0.02	3.39	−0.39
**63**	3.00	3.12	−0.12	3.28	−0.28
**64**	6.41	6.36	0.05	6.11	0.30
**65**	5.59	5.58	0.01	5.64	−0.05
**66**	3.72	3.72	0.00	3.62	0.09
**67**	3.00	2.98	0.02	3.18	−0.18
**68**	5.10	5.08	0.02	5.00	0.10
**69**	6.66	6.72	−0.06	6.59	0.07
**70**	6.82	6.78	0.05	6.98	−0.15
**71**	8.00	8.04	−0.04	8.00	0.00
**72**	8.00	7.98	0.02	8.05	−0.05
**Test Set**					
**73**	6.70	6.69	0.01	6.47	0.23
**74**	4.96	4.48	0.48	4.83	0.13
**75**	3.40	4.83	−1.43	6.52	−3.12
**76**	3.40	3.88	−0.49	2.80	0.60
**77**	6.52	6.53	−0.01	6.53	−0.01
**78**	6.00	6.27	−0.27	6.23	−0.23
**79**	5.51	4.31	1.20	3.45	2.06
**80**	3.97	5.20	−1.23	5.36	−1.39
**81**	6.52	6.64	−0.12	5.63	0.89
**82**	6.40	8.84	−2.45	11.19	−4.79
**83**	4.32	3.85	0.47	4.49	−0.17
**84**	3.70	4.96	−1.26	3.81	−0.11
**85**	6.77	5.48	1.29	6.26	0.51
**86**	3.00	3.38	−0.38	3.21	−0.21
**87**	3.00	4.21	−1.21	5.48	−2.48
**88**	3.00	2.05	0.95	5.24	−2.24
**89**	5.80	5.72	0.08	6.38	−0.58
**90**	3.82	4.21	−0.39	3.83	−0.01
**91**	3.00	4.95	−1.95	6.38	−3.38
**92**	6.82	7.54	−0.72	7.54	−0.71
**93**	8.00	7.70	0.30	7.62	0.38
**94**	7.10	7.63	−0.54	7.56	−0.46
**95**	8.54	9.44	−0.90	8.12	0.42
**96**	4.30	3.83	0.48	3.38	0.92
**97**	4.30	3.98	0.32	3.34	0.96

**Table 3:** Statistical parameters of the CoMFA models derived from Y-randomization tests.

Compounds	Q_cv_^2^	R_ncv_^2^	ONC	SEE	F value
**1**	−0.181	0.069	1	1.445	5.212
**2**	0.038	0.101	1	1.42	7.849
**3**	−0.181	0.055	1	1.456	4.109
**4**	−0.11	0.04	1	1.468	2.928
**5**	0.207	0.722	5	0.813	34.288
**6**	−0.225	0.135	1	1.393	10.968
**7**	−0.061	0.055	1	1.456	4.061
**8**	−0.046	0.063	1	1.45	4.685
**9**	−0.411	0.088	1	1.43	6.781
**10**	−0.019	0.069	1	1.446	5.156
**11**	−0.121	0.246	2	1.311	11.228
**12**	−0.023	0.058	1	1.454	4.272
**13**	−0.028	0.242	2	1.314	11.005
**14**	−0.164	0.059	1	1.453	4.389
**15**	0.012	0.087	1	1.431	6.681

**Table 4:** The docking results of the 97 compounds in the training and test sets.

Compounds	Fitness score	Orientation ^1^	Compound	Fitness score	Orientation ^1^
**Training Set**					
**1**	46.7447	Yes	**37**	67.605	Yes
**2**	65.4864	Yes	**38**	57.7153	No
**3**	69.988	Yes	**39**	50.9253	Yes
**4**	68.7748	Yes	**40**	69.5187	Yes
**5**	75.6942	Yes	**41**	73.0713	Yes
**6**	65.2663	Yes	**42**	61.2473	No
**7**	77.8612	Yes	**43**	57.4067	No
**8**	56.0559	Yes	**44**	68.8039	No
**9**	85.5617	Yes	**45**	79.0696	Yes
**10**	77.7628	Yes	**46**	51.0306	Yes
**11**	70.8855	Yes	**47**	55.4352	Yes
**12**	82.8049	Yes	**48**	57.8608	Yes
**13**	63.7736	Yes	**49**	59.4957	Yes
**14**	70.8527	No	**50**	65.029	Yes
**15**	56.3686	Yes	**51**	65.035	Yes
**16**	72.0652	No	**52**	56.6576	Yes
**17**	54.8263	Yes	**53**	64.2358	Yes
**18**	71.7425	Yes	**54**	60.8265	Yes
**19**	69.7473	Yes	**55**	51.5799	Yes
**20**	68.1959	Yes	**56**	58.7258	Yes
**21**	77.6519	Yes	**57**	56.3411	Yes
**22**	78.6929	Yes	**58**	66.6992	Yes
**23**	79.6385	Yes	**59**	57.0687	No
**24**	74.5361	Yes	**60**	42.5776	No
**25**	74.043	Yes	**61**	63.0642	Yes
**26**	79.7058	Yes	**62**	60.9584	Yes
**27**	90.9285	Yes	**63**	63.0642	Yes
**28**	88.8448	Yes	**64**	62.6583	Yes
**29**	59.6149	No	**65**	75.5489	Yes
**30**	69.544	No	**66**	52.7054	Yes
**31**	63.492	Yes	**67**	57.6862	Yes
**32**	45.5814	Yes	**68**	88.0785	Yes
**33**	60.9885	Yes	**69**	89.4423	Yes
**34**	67.8952	Yes	**70**	91.0785	Yes
**35**	75.3107	Yes	**71**	100.3275	Yes
**36**	67.5022	Yes	**72**	102.9909	Yes
**Test Set**					
**73**	70.0894	Yes	**85**	67.2837	Yes
**74**	71.9324	Yes	**86**	58.983	Yes
**75**	46.0479	No	**87**	55.1520	Yes
**76**	64.2422	Yes	**88**	47.7195	Yes
**77**	70.7896	Yes	**89**	65.82375	Yes
**78**	66.3318	Yes	**90**	56.9968	Yes
**79**	81.0177	Yes	**91**	57.2802	Yes
**80**	98.5388	Yes	**92**	93.4818	Yes
**81**	58.0293	Yes	**93**	94.082	Yes
**82**	64.0589	Yes	**94**	87.3304	Yes
**83**	61.4473	Yes	**95**	70.0355	Yes
**84**	58.8713	No	**96**	70.7513	No
**97**	81.0516	No			

^1^ “Yes” represents the pose of the substrates in the active pocket of MGMT protein being similar to the pose of the ligand in the crystal structure of MGMT (PDB entry: 1T38) and agreeing with the repairing mechanism of MGMT; “No” represents the opposite of “Yes”.

**Table 5:** Chemical structures and experimental activity values (pIC_50_) of the MGMT inhibitors.

Comp.	R_1_		R_2_	R_3_	R_4_	pIC_50_				
**Training Set**										
**1**	-OCH_3_		-NH_2_	H	H	3.46				
**2**	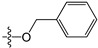		-NH_2_	H	H	6.70				
**3**	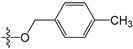		-NH_2_	H	H	6.70				
**4**	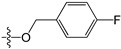		-NH_2_	H	H	6.70				
**5**	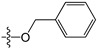		-NH_2_	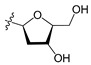	H	5.70				
**6**	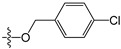		-NH_2_	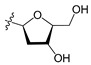	H	5.00				
**7**	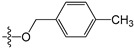		-NH_2_	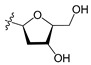	H	5.05				
**8**	-OCH_2_CH=CH_2_		-NH_2_	H	H	4.70				
**9**	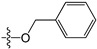		-NH_2_	-CH_2_COOCH_2_CH_3_	H	4.52				
**10**	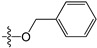		-NH_2_	-CH_2_C≡N	H	4.89				
**11**	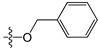		-NH_2_	-CH_2_CONH_2_	H	4.33				
**12** (*R/S*) ^1^	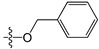		-NH_2_	-CH_2_CH(OH)CH_2_CH_3_ (*R/S*)	H	4.89				
**13**	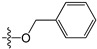		H	H	H	4.07				
**14**	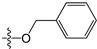		-NH_2_	-CH_2_COOCH_2_CH_3_	-	3.40				
**15**	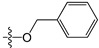		-NH_2_	-CH_2_CONH_2_	-	3.40				
**16** (*R/S*)	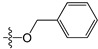		-NH_2_	-CH_2_CH(OH)CH_2_CH_3_ (*R/S*)	-	3.40				
**17**	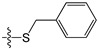		-NH_2_	H	H	3.40				
**18**	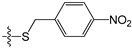		-NH_2_	H	H	3.40				
**19**	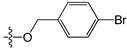		-NH_2_	H	H	6.52				
**20**	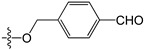		-NH_2_	H	H	6.30				
**21**	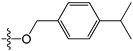		-NH_2_	H	H	6.30				
**22**	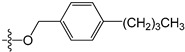		-NH_2_	H	H	5.40				
**23**	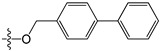		-NH_2_	H	H	6.52				
**24**	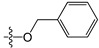		-NH_2_	-CHO	H	6.40				
**25**	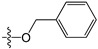		-NH_2_	-CH_3_	H	5.59				
**26** (*R/S*)	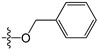		-NH_2_	-CH_2_CH(OH)CH_2_Cl (*R/S*)	H	4.74				
**27** (*R/S*)	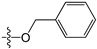		-NH_2_	-CH_2_CH(OH)CH_2_NHCH-(CH_3_)_2_ (*R/S*)	H	3.97				
**28** (*R/S*)	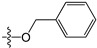		-NH_2_	-CH_2_CH(OH)CH_2_OCH-(CH_3_)_2_ (*R/S*)	H	5.15				
**29**	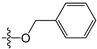		-NHCOCH_3_	H	H	4.62				
**30**	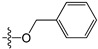		-NH_2_	-CH_3_	-	4.28				
**31**	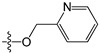		-NH_2_	H	H	4.24				
**32**	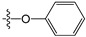		-NH_2_	H	H	3.52				
**33**	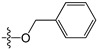		-NH_2_	H	-NH_2_	6.15				
**34**	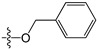		-NH_2_	H	-OH	6.52				
**35**	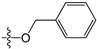		-NH_2_	H	-Br	7.10				
**36**	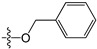		-OH	H	H	4.22				
**37**	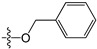		-OH	H	-OH	4.60				
**38**	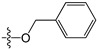		-NHCOCH_3_	H	-OH	4.19				
**39**	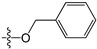		-NHCH_3_	H	H	3.80				
**40**	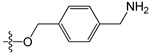		-NH_2_	H	H	6.82				
**41**	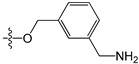		-NH_2_	H	H	6.96				
**42**	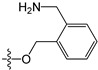		-NH_2_	H	H	4.28				
**43**	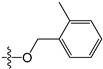		-NH_2_	H	H	3.91				
**44**	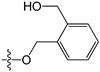		-NH_2_	H	H	3.81				
**45**	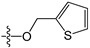		-NH_2_	H	H	8.52				
**46**	-OCH_2_CH_3_		-NH_2_	H	H	3.00				
**47**	-O(CH_2_)_2_CH_3_		-NH_2_	H	H	3.00				
**48**	-O(CH_2_)_3_CH_3_		-NH_2_	H	H	3.31				
**49**	-O(CH_2_)_2_CH(CH_3_)_2_		-NH_2_	H	H	3.00				
**50**	-O(CH_2_)_5_CH_3_		-NH_2_	H	H	3.26				
**51**	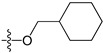		-NH_2_	H	H	3.00				
**52**	-OCH_2_CH(=CH_2_)CH_3_		-NH_2_	H	H	4.60				
**53**	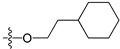		-NH_2_	H	H	3.26				
**54**	-OCH_2_CH(=CH_2_)CH_2_CH_3_		-NH_2_	H	H	4.80				
**55**	-OCH_2_CH(=CH_2_)CH_2_(CH_3_)_2_		-NH_2_	H	H	3.00				
**56**	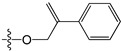		-NH_2_	H	H	4.11				
**57**	-OCH_2_C≡CH		-NH_2_	H	H	4.70				
**58**	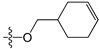		-NH_2_	H	H	3.00				
**59**	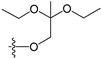		-NH_2_	H	H	3.00				
**60**	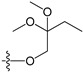		-NH_2_	H	H	3.00				
**61**	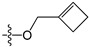		-NH_2_	H	H	6.26				
**62**	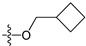		-NH_2_	H	H	3.00				
**63**	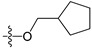		-NH_2_	H	H	3.00				
**64**	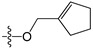		-NH_2_	H	H	6.41				
**65**	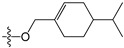		-NH_2_	H	H	5.59				
**66**	-OCH_2_COCH_3_		-NH_2_	H	H	3.72				
**67**	-OCH_2_COCH(CH_3_)_2_		-NH_2_	H	H	3.00				
**68**	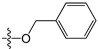		-NH_2_	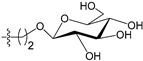	H	5.10				
**69**	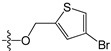		-NH_2_	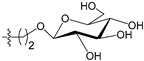	H	6.66				
**70**	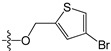		-NH_2_	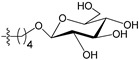	H	6.82				
**71**	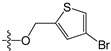		-NH_2_	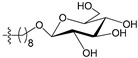	H	8.00				
**72**	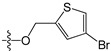		-NH_2_	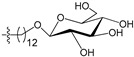	H	8.00				
**Test Set**										
**73**	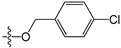		-NH_2_	H	H	6.70				
**74**	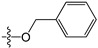		-NH_2_	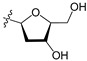	H	4.96				
**75**	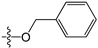		-NH_2_	-CH_2_C≡CH	-	3.40				
**76**	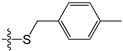		-NH_2_	H	H	3.40				
**77**	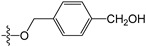		-NH_2_	H	H	6.52				
**78**	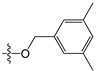		-NH_2_	H	H	6.00				
**79**	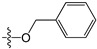		-NH_2_	-CH_2_OCOC(CH_3_)_3_	H	5.51				
**80** (*R/S*)	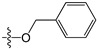		-NH_2_	-CH_2_CH(OH)CH_2_NH-C(CH_3_)_3_ (*R/S*)	H	3.97				
**81**	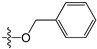		-NH_2_	H	-CH_3_	6.52				
**82**	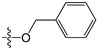		-NH_2_	H	-CF_3_	6.40				
**83**	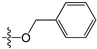		-F	H	H	4.32				
**84**	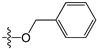		-N(CH_3_)_2_	H	H	3.70				
**85**	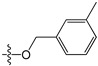		-NH_2_	H	H	6.77				
**86**	-O(CH_2_)_4_CH_3_		-NH_2_	H	H	3.00				
**87** (*R/S*)	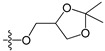 (*R/S*)		-NH_2_	H	H	3.00				
**88**	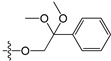		-NH_2_	H	H	3.00				
**89**	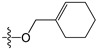		-NH_2_	H	H	5.80				
**90**	-OCH_2_COCH_2_CH_3_		-NH_2_	H	H	3.82				
**91**	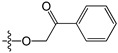		-NH_2_	H	H	3.00				
**92**	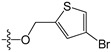		-NH_2_	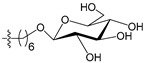	H	6.82				
**93**	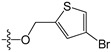		-NH_2_	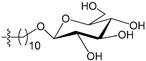	H	8.00				
**94**	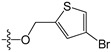		-NH_2_	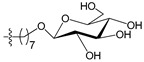	H	7.10				
**95**	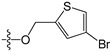		-NH_2_	H	H	8.54				
**96**	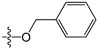		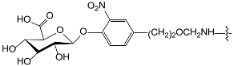	H	H	4.30				
**97**	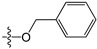		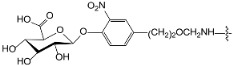	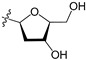	H	4.30				

## Supplementary Materials

Supplementary materials can be accessed at: http://www.mdpi.com/1420-3049/21/7/823/s1.
