# Supplementary material for: Identification of the Structural Features of Guanine Derivatives as MGMT Inhibitors Using 3D-QSAR Modeling Combined with Molecular Docking
Source: Molecules. 2016 Jun 23;21(7):823. doi: 10.3390/molecules21070823 (PMC6273773; doi:10.3390/molecules21070823)
Supplement: Supplementary file 1 [file molecules-21-00823-s001.pdf]

Supplementary Materials: Identification of the Structural Features of Guanine Derivatives as MGMT Inhibitors Using 3D-QSAR Modeling Combined with Molecular Docking

Guohui Sun, Tengjiao Fan, Na Zhang, Ting Ren, Lijiao Zhao and Rugang Zhong

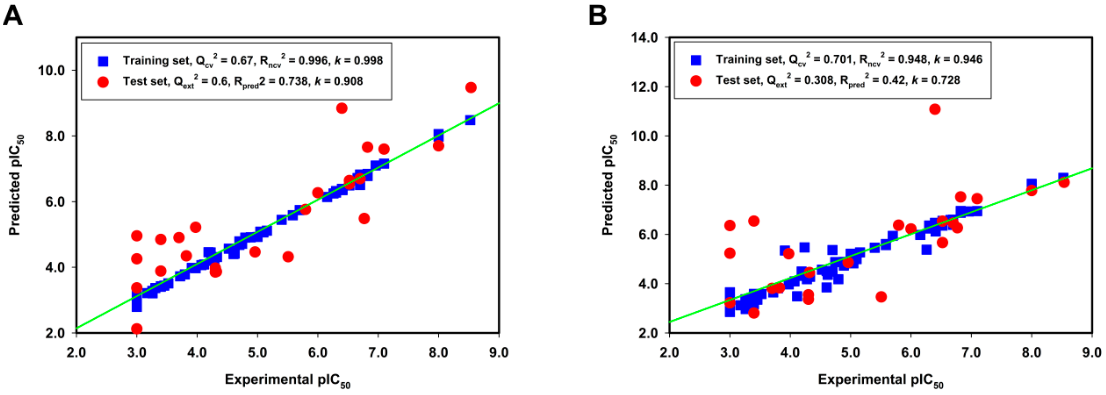

Figure S1. The linear correlation between the experimental and predicted pIC_{50} values for the training set (blue square) and the test set (red circle) based on (**A**) the CoMFA model and (**B**) the CoMSIA model derived from the ligand-based alignment method by using the *S*-isomers of chiral molecules and other molecules.

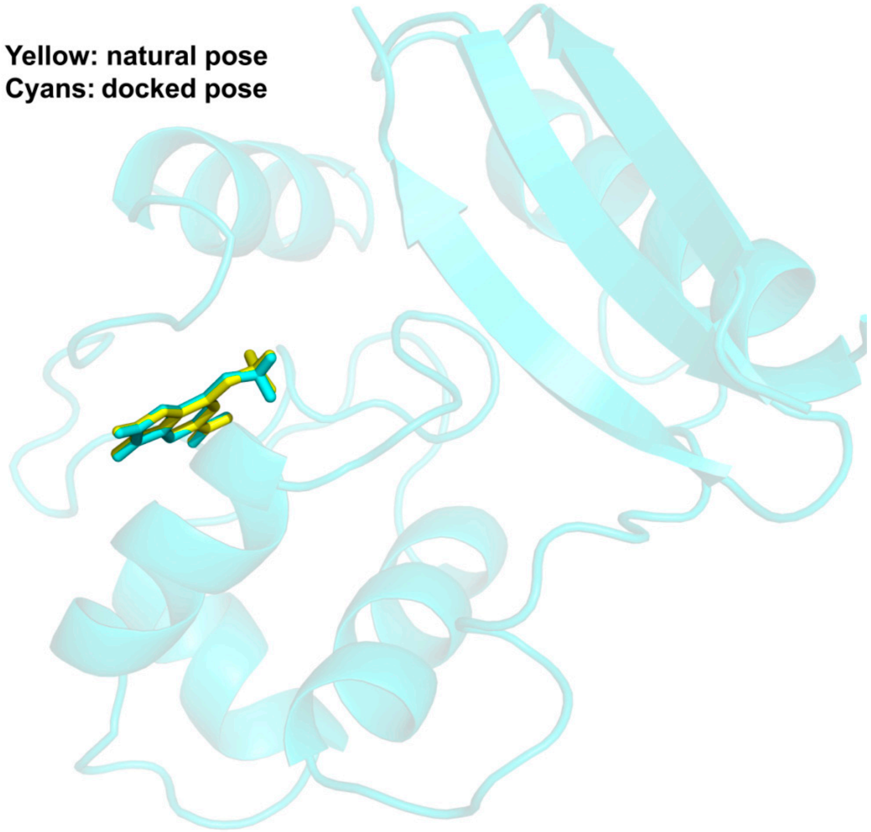

Figure S2. Overlap of the ligand between the docked pose (cyans) and the pose in protein-ligand crystal complex (yellow).

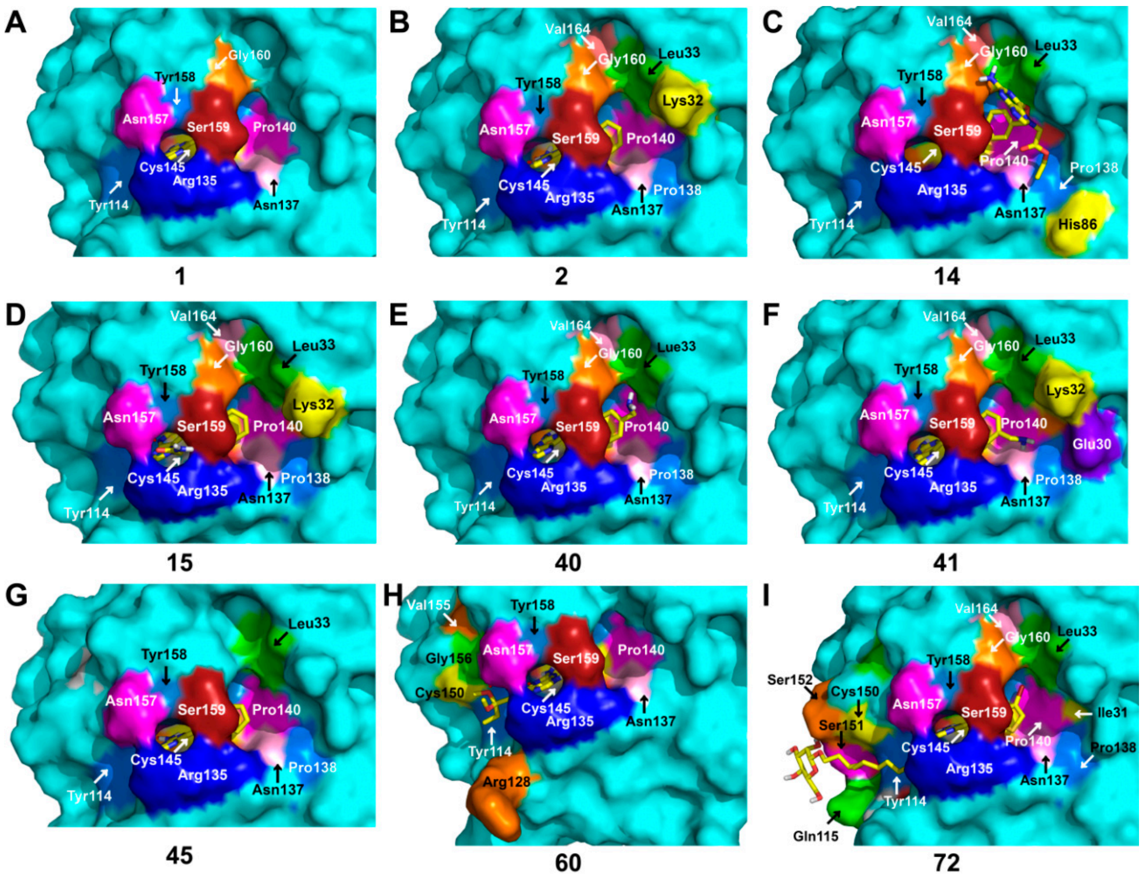

Figure S3. The ligand-binding surface of MGMT protein with the compounds described in Figure 4. The protein is displayed as solid surface with cyan. The ligands are presented in stick models with yellow representing carbon atom. The residues around the active pocket are displayed in different colors. All figures were generated using PyMOL software (Educational version; www.pymol.org; DeLano Scientific, San Carlos, CA, USA.

Table S1. Statistical parameters of the CoMFA and CoMSIA models derived from three alignment methods by using the *S*-isomers of chiral molecules and other molecules.[1]

Parameters [2]	Ligand-Based Alignment		DFT Optimization-Based Alignment		Docking-Based Alignment	
	CoMFA	CoMSIA[3]	CoMFA	CoMSIA [3]	CoMFA	CoMSIA [3]
Q_{cv}^2	0.67	0.701	0.498	0.498	0.168	0.401
ONC	8	13	4	4	4	5
R_{ncv}^2	0.996	0.948	0.718	0.693	0.697	0.766
SEE	0.088	0.384	0.829	0.807	0.903	0.743
F value	1090.24	77.0	43.245	38.525	38.031	42.486
**Field Distribution (%)**						
Steric	55.4	25.9	66.6	54.5	47.2	15.5
Electrostatic	44.6	45.1	33.4	–	52.8	46.3
Hydrophobic	–	–	–	–	–	38.2
HBD	–	13.5	–	45.5	–	–
HBA	–	15.5	–	–	–	–

[1] The CoMFA and CoMSIA models derived from three alignment methods were constructed by using the *S*-isomers of chiral molecules and other molecules. [2] Q_{cv}^2, ONC, R_{ncv}^2, SEE, F value, HBD and HBA are cross-validated correlation coefficient, optimal number of principal components, non-cross-validated correlation coefficient, standard error of estimate, F test value, hydrogen bond donor and hydrogen bond acceptor, respectively. [3] The parameters of CoMSIA models were derived from the combination of different fields that generates the highest Q_{cv}^2 value.

Table S2. Comparison of the experimental pIC_{50} values, predicted pIC_{50} values and residual values of the 97 compounds for CoMFA and CoMSIA models derived from the ligand-based alignment method by using the *S*-isomers of chiral molecules and other molecules.

**Compounds**	**Experimental pIC_{50}**	**CoMFA**		**CoMSIA**	
		**Predicted pIC_{50}**	**Residues**	**Predicted pIC_{50}**	**Residues**
**Training Set**					
**1**	3.46	3.44	0.02	3.35	0.11
**2**	6.70	6.51	0.19	6.39	0.31
**3**	6.70	6.61	0.09	6.43	0.27
**4**	6.70	6.83	−0.13	6.60	0.1
**5**	5.70	5.74	−0.04	5.94	−0.24
**6**	5.00	4.92	0.08	5.21	−0.21
**7**	5.05	5.08	−0.03	4.82	0.23
**8**	4.70	4.68	0.02	4.49	0.21
**9**	4.52	4.57	−0.05	4.57	−0.05
**10**	4.89	4.91	−0.02	4.88	0.01
**11**	4.33	4.31	0.02	4.28	0.05
**12**	4.89	4.91	−0.02	4.73	0.16
**13**	4.07	4.06	0.01	4.09	−0.02
**14**	3.40	3.41	−0.01	3.38	0.02
**15**	3.40	3.42	−0.02	3.36	0.04
**16**	3.40	3.42	−0.02	3.59	−0.19
**17**	3.40	3.41	−0.01	3.17	0.23
**18**	3.40	3.41	−0.01	3.27	0.13
**19**	6.52	6.59	−0.07	6.36	0.16
**20**	6.30	6.28	0.02	6.36	−0.06
**21**	6.30	6.32	−0.02	6.24	0.06
**22**	5.40	5.45	−0.05	5.46	−0.06
**23**	6.52	6.49	0.03	6.38	0.14
**24**	6.40	6.39	0.01	6.47	−0.07
**25**	5.59	5.59	0	5.57	0.02
**26**	4.74	4.71	0.03	4.88	−0.14
**27**	3.97	3.97	0	3.97	0
**28**	5.15	5.11	0.04	5.27	−0.12
**29**	4.62	4.41	0.21	4.36	0.26
**30**	4.28	4.24	0.04	4.28	0
**31**	4.24	4.19	0.05	5.47	−1.23
**32**	3.52	3.51	0.01	3.58	−0.06
**33**	6.15	6.14	0.01	5.98	0.17
**34**	6.52	6.52	0	6.55	−0.03
**35**	7.10	7.16	−0.06	6.95	0.15
**36**	4.22	4.45	−0.23	4.32	−0.1
**37**	4.60	4.40	0.2	4.51	0.09
**38**	4.19	4.46	−0.27	4.50	−0.31
**39**	3.80	3.78	0.02	3.81	−0.01
**40**	6.82	6.84	−0.02	6.88	−0.06
**41**	6.96	7.10	−0.14	6.93	0.03
**42**	4.28	4.21	0.07	4.18	0.1

**43**	3.91	3.98	−0.07	5.35	−1.44
**44**	3.18	3.21	−0.03	3.12	0.06
**45**	8.52	8.48	0.04	8.30	0.22
**46**	3.00	3.01	−0.01	3.26	−0.26
**47**	3.00	2.90	0.1	2.99	0.01
**48**	3.31	3.37	−0.06	3.31	0
**49**	3.00	3.02	−0.02	3.13	−0.13
**50**	3.26	3.28	−0.02	3.37	−0.11
**51**	3.00	2.79	0.21	3.12	−0.12
**52**	4.60	4.50	0.1	3.85	0.75
**53**	3.26	3.21	0.05	2.97	0.29
**54**	4.80	4.90	−0.1	4.18	0.62
**55**	3.00	3.05	−0.05	3.65	−0.65
**56**	4.11	4.09	0.02	3.49	0.62
**57**	4.70	4.79	−0.09	5.37	−0.67
**58**	3.00	3.17	−0.17	2.97	0.03
**59**	3.00	2.97	0.03	2.85	0.15
**60**	3.00	2.99	0.01	3.26	−0.26
**61**	6.26	6.25	0.01	5.38	0.88
**62**	3.00	3.03	−0.03	3.27	−0.27
**63**	3.00	3.13	−0.13	3.27	−0.27
**64**	6.41	6.35	0.06	6.12	0.29
**65**	5.59	5.58	0.01	5.61	−0.02
**66**	3.72	3.73	−0.01	3.65	0.07
**67**	3.00	2.97	0.03	3.20	−0.2
**68**	5.10	5.06	0.04	4.99	0.11
**69**	6.66	6.72	−0.06	6.59	0.07
**70**	6.82	6.78	0.04	6.95	−0.13
**71**	8.00	8.06	−0.06	8.01	−0.01
**72**	8.00	7.98	0.02	8.06	−0.06
			**Test Set**		
**73**	6.70	6.69	0.01	6.46	0.24
**74**	4.96	4.46	0.5	4.86	0.1
**75**	3.40	4.84	−1.44	6.54	−3.14
**76**	3.40	3.88	−0.48	2.80	0.6
**77**	6.52	6.53	−0.01	6.53	−0.01
**78**	6.00	6.27	−0.27	6.22	−0.22
**79**	5.51	4.32	1.19	3.46	2.05
**80**	3.97	5.21	−1.24	5.21	−1.24
**81**	6.52	6.64	−0.12	5.67	0.85
**82**	6.40	8.84	−2.44	11.08	−4.68
**83**	4.32	3.86	0.46	4.45	−0.13
**84**	3.70	4.90	−1.2	3.81	−0.11
**85**	6.77	5.48	1.29	6.26	0.51
**86**	3.00	3.37	−0.37	3.22	−0.22
**87**	3.00	4.25	−1.25	5.23	−2.23
**88**	3.00	2.12	0.88	5.23	−2.23

**89**	5.80	5.76	0.04	6.37	−0.57
**90**	3.82	4.35	−0.53	3.83	−0.01
**91**	3.00	4.95	−1.95	6.36	−3.36
**92**	6.82	7.66	−0.84	7.52	−0.7
**93**	8.00	7.70	0.3	7.78	0.22
**94**	7.10	7.60	−0.5	7.45	−0.35
**95**	8.54	9.47	−0.93	8.12	0.42
**96**	4.30	3.85	0.45	3.36	0.94
**97**	4.30	3.97	0.33	3.54	0.76
